# Cytoreductive surgery plus hyperthermic intraperitoneal chemotherapy improves survival of gastric cancer with peritoneal carcinomatosis: evidence from an experimental study

**DOI:** 10.1186/1479-5876-9-53

**Published:** 2011-05-07

**Authors:** Li Tang, Lie-Jun Mei, Xiao-Jun Yang, Chao-Qun Huang, Yun-Feng Zhou, Yutaka Yonemura, Yan Li

**Affiliations:** 1 Department of Oncology, Zhongnan Hospital of Wuhan University; Hubei Key Laboratory of Tumor Biological Behaviors & Hubei Clinical Cancer Study Center, Wuhan, 430071, P.R. China; 2 NPO Organization to Support Peritoneal Dissemination Treatment, Kishiwada, Osaka, Japan

## Abstract

**Background:**

Cytoreductive surgery (CRS) plus hyperthermic intraperitoneal chemotherapy (HIPEC) has been considered as a promising treatment modality for gastric cancer with peritoneal carcinomatosis (PC). However, there have also been many debates regarding the efficacy and safety of this new approach. Results from experimental animal model study could help provide reliable information. This study was to investigate the safety and efficacy of CRS + HIPEC to treat gastric cancer with PC in a rabbit model.

**Methods:**

VX2 tumor cells were injected into the gastric submucosa of 42 male New Zealand rabbits using a laparotomic implantation technique, to construct rabbit model of gastric cancer with PC. The rabbits were randomized into control group (n = 14), CRS alone group (n = 14) and CRS + HIPEC group (n = 14). The control group was observed for natural course of disease progression. Treatments were started on day 9 after tumor cells inoculation, including maximal removal of tumor nodules in CRS alone group, and maximal CRS plus heperthermic intraperitoneal chemoperfusion with docetaxel (10 mg/rabbit) and carboplatin (40 mg/rabbit) at 42.0 ± 0.5°C for 30 min in CRS + HIPEC group. The primary endpoint was overall survival (OS). The secondary endpoints were body weight, biochemistry, major organ functions and serious adverse events (SAE).

**Results:**

Rabbit model of gastric cancer with PC was successfully established in all animals. The clinicopathological features of the model were similar to human gastric PC. The median OS was 24.0 d (95% confidence interval 21.8 - 26.2 d ) in the control group, 25.0 d (95% CI 21.3 - 28.7 d ) in CRS group, and 40.0 d (95% CI 34.6 - 45.4 d ) in CRS + HIPEC group (*P *= 0.00, log rank test). Compared with CRS only or control group, CRS + HIPEC could extend the OS by at least 15 d (60%). At the baseline, on the day of surgery and on day 8 after surgery, the peripheral blood cells counts, liver and kidney functions, and biochemistry parameters were all comparable. SAE occurred in 0 animal in control group, 2 animals in CRS alone group including 1 animal death due to anesthesia overdose and another death due to postoperative hemorrhage, and 3 animals in CRS + HIPEC group including 1 animal death due to anesthesia overdose, and 2 animal deaths due to diarrhea 23 and 27 d after operation.

**Conclusions:**

In this rabbit model of gastric cancer with PC, CRS alone could not bring benefit while CRS + HIPEC with docetaxel and carboplatin could significantly prolong the survival with acceptable safety.

## Background

The loco-regional progression of gastric cancer usually results in peritoneal carcinomatosis (PC), characterized by the presence of tumor nodules of various size, number, and distribution on the peritoneal surface as well as malignant ascites, with very poor prognosis [1-5]. Patients with gastric PC face a dismal outcome, with a median survival of about 6 months [6].

Current treatments for such PC are systemic chemotherapy, best support care and palliative therapy. In order to tackle this problem, a new treatment modality called cytoreductive surgery (CRS) plus hyperthermic intraperitoneal chemotherapy (HIPEC) has been developed over the past 3 decades, taking advantages of surgery to reduce visible tumor burden, and regional hyperthermic chemotherapy to eradicate micrometastases [7-10]. Although many clinical studies have been performed to test and confirm the efficacy of this combined treatment approach, there is a lack of high quality evidence from phase III randomized prospective studies. In order to more objectively evaluate such treatment, it is necessary to study this treatment modality under experimental conditions, in which most of the confounding factors could be well controlled. In this respect, suitable animal models of PC are indispensable platforms. Small animal models of PC have been established, including mouse models and rat models [11-18]. In most of these animal models, cancer cells are injected directly into the peritoneum, which will result in widespread PC in due time. Such models have been used to test various treatment modalities, including CRS and HIPEC, either alone or in combination, producing valuable information on the validity of different therapies. The small body size and delicate hemodynamic conditions are limiting factors for complex surgical interventions. Large animal models of PC might be more suitable for extensive surgical treatment. Therefore, it is necessary to establish large animal model of PC from gastric cancer for experimental studies to test extensive CRS and HIPEC.

In our previous study [19], we have established a stable rabbit model of gastric cancer with PC by injecting VX2 cancer cells into the submucosal layer of the stomach. The model is characterized by typical ulcerative gastric cancer with progressive PC, making it more suitable for surgical interventional studies to evaluate CRS and HIPEC against gastric PC.

This rabbit model of gastric cancer with PC has provided us with suitable platform to evaluate different therapeutic approaches against PC. This study was designed to evaluate the efficacy and safety of CRS + HIPEC for the treatment of this large animal model of gastric PC, so as to provide support to clinical application.

## Methods

### Animals

Forty two male New Zealand white rabbits, body weight between 1.8-2.9 kg (Median 2.0 kg), were obtained from Animal Biosafety Level 3 Laboratory at the Animal Experimental Center of Wuhan University (Animal Study Certificate SCXK 00002826). The animals were individually housed and allowed free access to standard laboratory food and water as well as 12 h of light and dark cycle per day. The animal study protocol was approved by the Animal Welfare Committee of the Center.

### Construction of rabbit model of VX2 gastric carcinoma with PC

Rabbit VX2 carcinoma was used to establish gastric cancer with PC in this study. The animals were anesthetized by ear vein injection of 1% pentobarbital sodium (30 mg/kg). The abdominal skin was cleaned and disinfected. Tumor cells were injected into the stomach submucosa layer to construct rabbit models of PC as described previously [19]. Briefly, a midline incision of 3 cm long was made beginning 2 cm below the xyphoid and the upper abdomen was open. The stomach was exposed, 0.1 ml of tumor cells (5 × 10^10 ^vial cells/L) was injected into the submucosal layer of the stomach, through the serosal layer and the muscle layer, the injection site was pressed for 1 min to keep the injected tumor cells in place, and the abdomen was closed with a double layer 3-0 vicryl interrupted suture. After tumor inoculation, Penicillin G at the dose of 100,000 IU/d was intramuscularly injected to each animal for 3 d.

### Randomization and treatment

**Figure 1:**
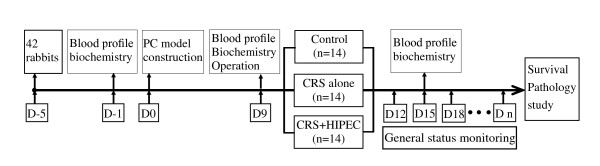
**The study protocol**. After construction of PC model of gastric cancer, 42 New Zealand white rabbits were randomized into 3 groups with 14 rabbits per group, and the effects of CRS and CRS + HIPEC were investigated. D, day; PC, peritoneal carcinomatosis; CRS, cytoreductive surgery; HIPEC, hyperthermic intraperitoneal chemotherapy.

When animal model construction has been confirmed successful on day 9 after operation, these rabbits were randomized into 3 groups according to a computer generated randomize number, 14 animals in each group (Figure 1).

The control group was observed for natural course of disease progression without any intervention.

For CRS alone group, CRS was performed on d 9 after tumor cells inoculation. Rabbits were given 1% pentobarbital sodium (30 mg/kg) intravenously for anesthesia. The abdominal skin was cleaned and disinfected. The abdominal exploration was performed through a midline incision of 8 cm long beginning 1 cm below the xyphoid. Once the abdominal wall was open, detailed evaluation of the PC was conducted in different regions including the parietal peritoneum, visceral peritoneum, the omentum, stomach, liver, spleen, small intestine, colon, bladder and other pelvic tissues. Thereafter, maximal CRS was performed including a routine omentectomy, and optimal removal of tumor nodules. Unresectable tumors were cauterized. The gastric tumor itself, however, was not removed but treated by injection of absolute alcohol. After completion of CRS the abdominal wall was closed in 2 layers using 3-0 Vicryl constinuous sutures.

For CRS + HIPEC group, maximal CRS was performed on d 9 in the same fashion as in the CRS alone group, which was immediately followed by HIPEC just before the closure of abdominal cavity. Open HIPEC was performed, as this open technique was believed to provide optimal thermal homogeneity and spatial diffusion [20,21], with 250 mL of heated saline containing 10 mg of docetaxel (Wanle Pharmaceutical Co., Ltd. Shenzhen, China.) and 40 mg of carboplatin (Qilu Pharmaceutical Co., Ltd. Shandong, China.) for each animal. The abdominal cavity was rinsed twice with 250 mL of normal saline preheated to 42.0°C and perfusion tube was placed in pelvic cavity just before HIPEC. The perfusion equipment consisted of a miniature heat exchanger and a roller pump, allowing perfusion with a variable dynamic flow of 6 - 12 ml/min. An inflow catheter was inserted into the upper abdomen between the hepatic and diaphragmatic surface and an outflow catheter was placed at the pelvic floor. The perfusion solution was heated to 42.0 ± 0.5°C and infused into the peritoneal cavity at a rate of 10 ml/min through the inflow tube introduced from the automatic perfusion pump. The perfusion in the peritoneal cavity was stirred manually to make equal spatial distribution. The temperature of the perfusion solution in peritoneal space was kept at 42.0 ± 0.5°C and monitored using a thermometer on real time. The total HIPEC time was 30 min, after which the perfusion solution in the abdominal cavity was removed.

Twenty min before surgery, 100 ml of 0.9% NaCl solution with 1 g of ceftriaxone powder, 2 ml of 10% potassium chloride solution and 20 ml of 50% glucose solution was infused intravenously for rehydration, energy support and infection control in both the CRS alone group and the CRS + HIPEC group. Such treatment was continued for 3 d.

### Animal observation and disease course monitoring

The general status of the animals was daily recorded in a standard form. For pathological studies, euthanasia was performed on the rabbits by overdose injection of 1% pentobarbital sodium through the ear vein. Post mortem pathological examinations included gross pathology such as tumor size and distributions; local tumor features of gastric cancer such as ulcer formation, obstruction and perforation; special features of peritoneal carcinomatosis such as bloody ascites, discrete or confluent tumor nodules on the peritoneum, omentum cake and intestinal obstructions; metastases to major organs such as the liver, adrenal glands, pancreas and the lungs.

For laboratory studies, 5 ml of blood was harvested from ear vein on the day before tumor cells inoculation as the baseline, on the day of surgery, and on d 8 after surgery. The samples were used for routine peripheral blood test, liver and kidney functions tests and biochemical tests.

### Statistical Analysis

The primary endpoint was overall survival (OS) in each group, defined as the time interval form animal model construction to animal death due to any cause, including cancer progress. The secondary endpoints were body weight, biochemistry, major organ functions and serious adverse events (SAE), which was defined as severe local and/or systemic infection or death due to the procedure.

In our previous study to construct this animal model, we learned that the median survival of this gastric PC model is about 3 weeks [19]. Therefore, we calculated the sample size of this study based on this information. This trial was designed to detect at least a 30% absolute difference in OS. With a statistical power of 90% to detect such difference at 5% significance level, at least 12 animals were required in each group. Taking into consideration of unexpected events during the performance of the study, we enlarged the sample size to 14 animals in each group. Categorized variables in the two groups were compared by chi square test or Fisher's exact test. The numerical data were directly recorded, and the category data were recorded into different categories. The Kaplan-Meier method was used to compare the survival, with log rank test. Data were analyzed using the Statistical Package for Social Sciences (SPSS Inc., Chicago, Illinois, USA), version 13.0 with 2-sided *P *< 0.05 as statistically significant.

## Results

### Histopathological characteristics of PC

Rabbit gastric cancer PC model was established in all animals (100%, 42/42). Nine days after tumor cells inoculation, many small, hard and transparent tumor nodules developed on the greater omentum, and typical ulcerative cancer about 0.5 cm in diameter formed on the antrum of the stomach. No ascites was observed. No obvious PC was found in other regions. There were no differences in the PC severity among three groups. This could be equivalent to clinical stage I peritoneal carcinomatosis by Gilly criteria [6].

Typical ulcerative cancer with PC was observed in post mortem pathological examinations of rabbits in control group. The stomach wall was totally invaded by the tumor to create cancer ulcer encased by confluent nodules on the greater omentum, forming a big tumor block. The abdominal wall and diaphragm were totally invaded by the tumor. Many tumor nodules formed on the intestinal wall, the mesentery and the retroperitoneum. Bloody ascites could be more than 100 ml. All the features are similar to the clinicopathologic characteristics of gastric cancer with PC in patients.

### Body weight changes

**Figure 2:**
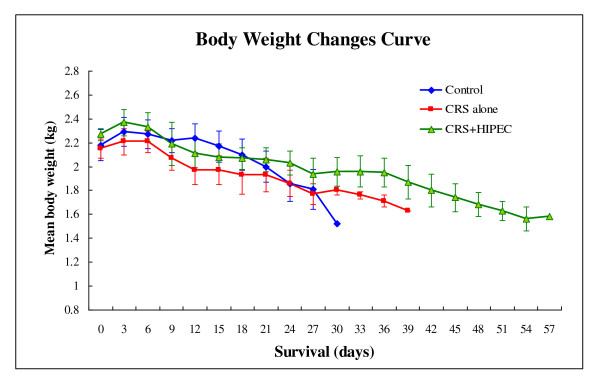
**Body weight changes in 3 groups of rabbits**. Compared with control and CRS groups, CRS + HIPEC group experienced slower body weight loss, although the differences among the 3 groups did not reach statistical significance.

The body weight of each animal was recorded every 3 d. No significant differences were found in initial body weight of 3 groups before the treatment. Perioperative body weight decreased in all groups because of the overnight fasting. In the control group, the body weight recovered once food intake was resumed but again decreased progressively till the study endpoint. In the 2 treatment groups, postoperative body weight decreased considerably during the first 3 d after surgery and then decrease became gentle along with the increased food intake in the following 5 d in 2 treatment groups. Thereafter, body weight decreased progressively again till the study endpoint in CRS alone group, while body weight could be maintained or slightly increased for the following 20 d in CRS + HIPEC group and decreased slowly till the study endpoint (Figure 2).

### Blood profile changes

At the baseline, on the day of surgery and on day 8 after surgery, the peripheral blood cells counts, liver and kidney function tests, and biochemistry parameters were all comparable (Table 1).

**Table 1:** Blood routine tests and biochemical test results

		Range (median)			
Parameters		Control (n = 14)	CRS (n = 14)	CRS+HIPEC (n = 14)	*P*
**Peripheral blood tests**					
	A	139~156 (149)	129~139 (131)	124~141 (131)	NS
HGB (G/L)	B	112~130 (128)	102~137 (121)	104~30 (126)	NS
	C	78~135 (117)	62~123 (100)	80~103 (92)	NS
	A	6.55~7.30 (6.77)	6.19~6.80 (6.77)	5.98~6.48 (6.29)	NS
RBC (× 10^9^/L)	B	5.19~6.18 (5.76)	5.26~6.54 (5.60)	4.87~6.37 (6.14)	NS
	C	4.27~6.14 (5.26)	2.26~5.76 (5.30)	3.97~4.70 (4.34)	NS
	A	4.0~10.1 (6.5)	7.4~9.9 (8.8)	4.2~8.7 (4.7)	NS
WBC (× 10^9^/L)	B	6.5~11.3 (8.0)	4.8~10.3 (9.1)	7.1~9.4 (9.2)	NS
	C	7.7~18.2 (9.2)	3.2~8.3(4.9)	10.0~8.3 (9.2)	NS
	A	1.3~3.8 (2.7)	2.2~5.9 (4.5)	2.9~3.8 (3.2)	NS
Neu (× 10^9^/L)	B	1.8~4.3 (3.8)	1.4~8.4 (3.6)	2.6~3.8 (3.1)	NS
	C	1.1~4.8 (3.0)	0.7~4.1 (2.0)	4.2~4.8 (4.5)	NS
	A	169~385 (320)	158~410 (267)	94~415 (319)	NS
PLT (× 10^9^/L)	B	68~434 (231)	103~398 (232)	232~682 (360)	NS
	C	302~663 (324)	12~59 (27)	36~426 (231)	NS
**Liver function tests**					
	A	15~24 (22)	34~44 (35)	15~24 (22)	NS
AST (U/L)	B	28~91 (75)	65~72 (66)	36~8 (69)	NS
	C	27~30 (29)	14~22 (20)	15~19 (17)	NS
	A	28~43 (36)	65~72 (66)	36~81 (69)	NS
ALT (U/L)	B	12~41 (26)	16~38 (26)	19~89 (29)	NS
	C	42~131 (50)	54~90 (85)	71~95 (83)	NS
	A	60.7~77.0 (66.1)	62.8~66.5 (66.0)	58.4~66.3 (63.8)	NS
TP (g/L)	B	58 .0~69.9 (62.3)	56.5~69.6 (62.8)	53.2~71.5 (59.9)	NS
	C	61.1~65.6 (62.8)	46.3~63.7 (55.8)	50.2~57.8 (54.0)	NS
	A	37.3~41.2 (39.6)	36.6~41.8 (40.6)	32.0~41.2 (39.6)	NS
ALB (g/L)	B	35.0~41.4 (37.1)	31.3~39.8 (35.8)	32.5~38.4 (34.9)	NS
	C	34.6~38.9 (36.3)	26.4~37.4 (30.9)	30.7~32.8 (31.8)	NS
	A	23.4~35.8 (26.6)	24.8~26.3 (25.7)	26.4~32.6 (26.6)	NS
GLB (g/L)	B	22.2~28.5 (25.5)	24.4~32.2 (27.3)	19.7~33.1 (25.2)	NS
	C	23.9~29.3 (26.5)	19.9~26.3 (24.9)	19.5~25.0 (22.3)	NS
	A	126~190 (159)	127~248 (147)	159~186 (172)	NS
ALP (U/L)	B	78~145 (98)	45~177 (86)	51~133 (89)	NS
	C	73~118 (80)	58~114 (79)	52~114 (83)	NS
**Renal function tests**					
	A	6.24~15.08 (6.95)	6.47~7.68 (7.47)	7.28~8.44 (8.16)	NS
BUN (mmol/L)	B	8.83~14.77 (12.24)	0.59~16.64 (10.18)	6.82~14.94 (7.92)	NS
	C	5.45~6.45 (6.27)	5.33~7.07 (6.61)	4.83~6.45 (5.64)	NS
	A	81.0~121.2 (86.5)	80.6~99.2 (85.4)	81.0~95.3 (83.7)	NS
Cr (μmol/L)	B	75.0~99.0 (94.9)	70.4~107.4 (88.8)	85.8~97.8 (91.6)	NS
	C	67.3~85.6 (75.7)	60.3~69.9 (62.2)	65.8~66.9 (66.4)	NS
**Electrolytes**					
	A	4.10~18.97 (4.66)	3.52~4.72 (4.21)	3.99~10.97 (4.30)	NS
K+ (mmol/L)	B	7.34~27.13 (10.44)	4.44~11.09 (6.29)	4.34~12.29 (4.74)	NS
	C	5.14~5.91 (5.18)	4.98~6.08 (5.14)	5.31~6.32 (5.82)	NS
	A	139.1~148.7 (145.3)	142.8~148.8 (144.2)	142.2~145.3 (144.7)	NS
Na+ (mmol/L)	B	124.5~146.4 (140.4)	137.3~148.5 (141.65)	133.6~146.4 (141.1)	NS
	C	133.2~138.7 (133.5)	132.9~138.9 (135.3)	133.8~134.3 (134.1)	NS
	A	99.8~121.2 (102.6)	101.9~107.2 (103.5)	100.5~110.2 (102.1)	NS
Cl^- ^(mmol/L)	B	94.6~103.9 (96.6)	96.6~107.7 (101.1)	97.0~106.6 (100.8)	NS
	C	100.0~104.1 (103.7)	102.2~106.6 (104.7)	103.8~104.2 (104.0)	NS
	A	3.11~3.77 (3.39)	3.05~3.18 (3.12)	3.18~3.69 (3.63)	NS
Ca++ (mmol/L)	B	2.80~4.04 (3.70)	3.41~3.96 (3.71)	3.32~3.96 (3.66)	NS
	C	3.71~4.10 (3.80)	3.19~3.56 (3.45)	3.48~3.52 (3.50)	NS

RBC: red blood cells; WBC: white blood cells; HGB: hemoglobin; Neu: neutrophils count; PLT: platelets counts; ALT: alanine transaminase; AST: aspartate aminotransferase; A: At baseline; B: On the day of surgery; C: On d 8 after surgery; TP: total protein; ALB: albumin; GLB: globulin; BUN: blood urea nitrogen; Cr: creatinine.

### Survival

**Figure 3:**
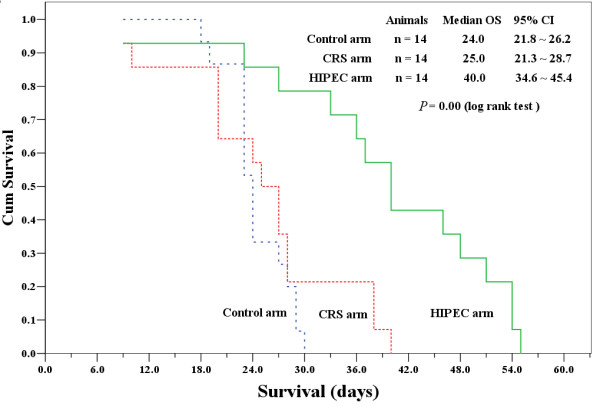
**Kaplan-Meier survival curves for control, CRS alone, and CRS + HIPEC groups**. Compared with CRS only or control group, CRS + HIPEC could extend OS by at least 15 d (60%). (*P *= 0.00, log rank test)

The animals in the control group did not receive any active surgical treatment, and only observed for natural history of disease progression. For animals in both CRS and CRS+HIPEC groups, complete cytoreduction was achieved either by surgical resection or cauterization for the peritoneal carcinomatosis, leaving no observable tumor nodules in the peritoneal cavity. The gastric tumor itself, however, was not removed but treated by injection of absolute alcohol. The median OS was 24.0 d (95% CI 21.8 - 26.2 d) in the control group, 25.0 d (95% CI 21.3 - 28.7 d) in CRS group, and 40.0 d (95% CI 34.6 - 45.4 d) in CRS + HIPEC group (*P *= 0.00, log rank test). Compared with CRS only or control group, CRS + HIPEC could extend OS by at least 15 d (60%) (Figure 3).

### Postmortem pathological examinations

Euthanasia was performed on the moribund rabbits by overdose injection of 1% pentobarbital sodium through the ear vein. Detailed information on postmortem pathological examinations was listed in Table 2.

**Table 2:** Results of post mortem pathological study in 3 groups*

	Control (n = 14)	CRS (n = 12)*	CRS+HIPEC (n = 13)§	*P*
Ulcerative gastric cancer	100%	100%	100%	NA
Pyloric obstruction	100%	100%	100%	NA
Gastric perforation	28.6%	8.3%	8.3%	*P *= 0.246
Greater omentum cake	100%	removed	removed	NA
Liver metastases	100%	96.7%	38.5%	***P *= 0.000**
Pulmonary metastases	0.0%	8.3%	53.8%	***P *= 0.000**
Cancerous diaphragm	100%	100%	53.8%	***P *= 0.000**
Upper abdominal wall cancer	100%	100%	84.6%	*P *= 0.128
Small intestine & mesentery seeding	100%	100%	61.5%	***P *= 0.002**
Adrenal gland metastases	100%	100%	23.5%	***P *= 0.000**
Kidney capsule invasion	100%	75.0%	30.8%	***P *= 0.000**
Retroperitoneum metastases	100%	100%	76.9%	***P *= 0.038**
Pelvic seeding	100%	100%	69.2%	***P *= 0.009**
Urine retention	57.1%	75.0%	61.5%	*P *= 0.641
Bloody ascites	100%	100%	38.5%	***P *= 0.000**

* Two animals were excluded in CRS group, including 1 death due to anesthesia overdose on d 9 and another death due to postoperative hemorrhage on d 10.

§ One animal in CRS+HIPEC group was excluded due to anesthesia overdose death on d 9.

NA: not applicable.

### Severe Adverse Events

**Figure 4:**
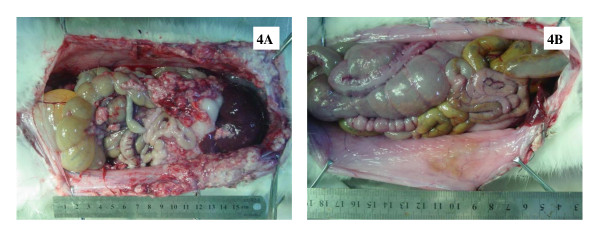
**On day 27, post mortem pathological examinations of a rabbit in CRS group (2A) and a rabbit in CRS + HIPEC group (2B)**. In CRS group, widespread PC recurrence was evident even after sytoreductive surgery. In the CRS + HIPEC group, hyperthermic chemoperfusion significantly retarded PC recurrence.

SAE occurred in 0 animal in control group, 2 animals in CRS alone group including 1 death due to anesthesia overdose (OS = 9 d) and another death due to postoperative hemorrhage (OS = 10 d), and 3 animals in CRS + HIPEC group including 1 death due to anesthesia overdose (OS = 9 d), and 2 deaths due to diarrhea 23 and 27 d after operation. A direct comparison in gross pathology on d 27 of a rabbit in CRS group (Figure 4A) and a rabbit in CRS + HIPEC group (Figure 4B) showed significant differences in PC severity.

## Discussion

This study has provided new evidence to support CRS + HIPEC to treat gastric PC. Compared with control group and CRS alone group, the CRS + HIPEC group could have an additional survival gain of at least 15 d (60%). In addition to such significant survival benefit, other improvements have also been observed, including body weight, PC severities, ascites, liver and kidney functions, and blood electrolytes.

This study also suggests that in established gastric PC, simply performing CRS may not bring survival benefit. The animals in the CRS group had a median survival of 25 d, which is not statistically different from 24 d in the control group.

PC has been increasingly recognized as an important clinical problem and increasing efforts have been devoted to investigating the mechanism and coping strategies against this disease. Clinical trials in selected gastric or colorectal PC patients have provided evidence in favor of CRS + HIPEC for these patients, and the only phase III prospective randomized trial in colorectal PC patients reported a median survival advantage of 70% gain in overall survival (22.4 months in the CRS + HIPEC group *VS *12.6 months with standard palliative care alone) [22]. The encouraging results by Yonemura [8] and Glehen [9] in gastric PC provided more compelling evidence to support this combined treatment modality. Nevertheless, controversies regarding the usefulness and value of such approach remain [23,24]. It seems unlikely that this issue will be resolved shortly in randomized clinical trials. Therefore, it is necessary to study the treatment modality under experimental conditions, in which most of the confounding factors could be well controlled for more objective evaluation of HIPEC.

In recent years, increasing number of animal models of PC have been intensively studied, including nude mouse model of gastric cancer PC constructed by implanting human gastric cancer cells [25-28]; mouse colon cancer PC model constructed by injecting colon cancer cells into the abdominal cavity of Balb/C mice [29]; rat colon carcinoma PC models constructed through injecting CC531 colon carcinoma cells into the abdominal cavity of Wag/Rij rats [15,30-33] or injecting syngeneic colon adenocarcinoma cells (DHD/K12/TRb) into the abdominal cavity of athymic BD IX/HansHsd rats [14,18,34,35]; murine xenograft PC model of appendiceal mucinous adenocarcinoma constructed by implanting human appendiceal neoplasms into the peritoneal cavity of homozygous nude mice [36]; mouse ovarian cancer PC model constructed through injecting human serous or epithelial ovarian cancer cells into the abdominal cavity of mice [37-39] or injecting murine ovarian surface epithelial cells (ID8 cells) under ovarian bursa of C57BL6 mice [39].

Compared with the small animal PC models, our rabbit model of gastric PC is the first large animal PC model, more suitable for complex surgical interventional studies such as CRS + HIPEC. In addition, this model reproduces the whole pathological process from the primary gastric cancer to the development of PC, resembling the complete clinico-pathological features of human gastric PC.

To our knowledge, there have been 3 reports in the literature on the efficacy of CRS + HIPEC in experimental animal models of PC. Klaver et al [34] used the rat colonic carcinoma PC model to test whether the addition of HIPEC to CRS is essential for survival benefit. The rats were randomized into 3 treatment groups of 20 rats each, CRS alone, CRS + HIPEC (mitomycin 15 mg/m^2 ^at 42.0°C for 90 min) and CRS + HIPEC (mitomycin C 35 mg/m^2 ^at 42.0°C for 90 min). The CRS + HIPEC achieved a significant survival gain of over 120% (the median survival of 43, 75 and 97 d, *P *< 0.01). Pelz et al [15] used similar rat colonic carcinoma PC model to investigate HIPEC. After 10 d of tumor cells inoculation, the rats were randomized into 3 groups of 6 animals each, control, HIPEC (mitomycin C 15 mg/m^2 ^at 40.5 - 41.5°C for 90 min), and normathermic intraperitoneal chemotherapy (mitomycin C 10 mg/m^2 ^i.p.). Although the study did not report the overall survival, the HIPEC group did have significantly smaller tumor weight, fewer tumor nodules, decreased cancer index and better clinical complete response rate, compared with control or normathermic ip mitomycin treatment alone. In a similar study on rat colon cancer PC model, Raue et al [36] found that only CRS + HIPEC with MMC 15 mg/m^2 ^at 41.2 - 42.3°C for 60 min could result in significant reduction in tumor weigh and PC index. Again this study did not report on the overall survival.

## Conclusions

In summary, this study on the first large animal model of gastric PC has proved that CRS + HIPEC could indeed bring survival benefit with acceptable safety, providing evidence to support this combined strategy to treat selected patients of gastric cancer with PC.

## Competing interests

The authors declare that they have no competing interests.

## Authors' contributions

YLI conceived, designed and partly conducted the study. LT, LJM, CQH and XJY conducted the study and drafted the manuscript. YFZ and YY provided technical support. All authors have read the approved the final manuscript.
